# Ulk4, a Newly Discovered Susceptibility Gene for Schizophrenia, Regulates Corticogenesis in Mice

**DOI:** 10.3389/fcell.2021.645368

**Published:** 2021-06-21

**Authors:** Ling Hu, Yi Chen, Cui-Ping Yang, Ying Huang, Ning-Ning Song, Jia-Yin Chen, Yu-Ling Sun, Yu-Qiang Ding, Bing Lang

**Affiliations:** ^1^State Key Laboratory of Medical Neurobiology and MOE Frontiers Center for Brain Science, Institutes of Brain Science, Fudan University, Shanghai, China; ^2^Key Laboratory of Arrhythmias, Ministry of Education, East Hospital, and Department of Anatomy and Neurobiology, Tongji University School of Medicine, Shanghai, China; ^3^Department of Laboratory Animal Science, Fudan University, Shanghai, China; ^4^National Clinical Research Centre for Mental Health, Department of Psychiatry, The Second Xiangya Hospital, Central South University, Changsha, Hunan, China

**Keywords:** schizophrenia, Ulk4, corticogenesis, knockout mice, neural progenitors

## Abstract

Schizophrenia (SCZ) is a chronic and severe mental disease that affects around 1% of the population. The precise etiology of SCZ still remains largely unknown, and no conclusive mechanisms are firmly established. Recent advances in epidemiological and clinical investigation support an overwhelmingly strong neurodevelopmental origin for SCZ. Here, we demonstrated that Unc-51-like kinase 4 (Ulk4), a novel risk factor for major mental disorders including schizophrenia, is involved in the corticogenesis. Deletion of Ulk4 in mice led to significantly thinner layers of II–III, and V in the cerebral cortex, which was confirmed in conditional Ulk4 deletion mice achieved by Cre-loxp strategy. This abnormality might be caused by decreased intermediate neural progenitors and increased apoptosis. Thus, our data suggest that Ulk4 manipulates the behaviors of neural progenitors during brain development and, when functionally defective, leads to the reduction of specific cortical layers. This anomaly may increase predisposition to a range of neurodevelopmental disorders, including SCZ.

## Introduction

Schizophrenia (SCZ) is a devastating brain disorder that affects approximately 1% of the population worldwide. It imposes substantial burden of morbidity and mortality and is often associated with an average 20–25 years reduction in life span. Despite strenuous research, the underlying neurobiology of SCZ has not been clearly established. Current treatments are mostly palliative and do not change the overall prognosis of the patients.

The root reasons for SCZ are complex, but heritability is considerably high (60–80%) (Camargo et al., 2007). Most of these brain-specific molecular and genetic findings, together with epidemiological evidence, all support an overwhelmingly strong neurodevelopmental origin for SCZ (Rapoport et al., 2005). It has been proposed that various genetic lesions can have detrimental effects as early as in the first or early second trimester (Fatemi and Folsom, 2009) and, as a result, can disturb the cellular architecture and synaptic connectivity of the developing brain of human embryos. These insults are believed to, alone or in combination, profoundly undermine brain maturation, which increases predisposition toward the occurrence of SCZ in late adolescence (Insel, 2010).

It is essential to understand how these genetic lesions mislead the formation of neural circuitry or mistune the brain function accordingly. However, high heritability has not been translated into the successful identification of candidate genetic lesions. Early genome-wide or candidate gene studies searching for common variants associated with SCZ are mostly disappointing, either because of poor replication or failure to detect genome-wide significance in large-scale studies. As an example, a recent large-scale genome-wide association study (GWAS) by the Psychiatric Genomics Consortium has identified 108 top genomic loci associated with SCZ, which, however, only explain 3.4% of the variance in risk profiles (Schizophrenia Working Group of the Psychiatric Genomics, 2014). Therefore, there remain many genetic variants and associated mechanisms for which data are missing.

Increasing evidence has suggested that aberrant brain architecture and function stem from the dysfunctional proliferation and differentiation of neural stem cells (NSCs)—a major convergent point for neurodevelopmental disorders including SCZ (Ernst, 2016). Indeed, a major focus in current psychiatric genetics research is to investigate the function of key variants in brain development and find out either unique downstream molecules or convergent signaling pathways. This may offer novel insights to better interpret the biology of these refractory disorders and, more importantly, for translation into early diagnosis and intervention.

Based on the supportive datasets from the International Schizophrenia Consortium and Decode, we recently have identified Unc-51-like kinase 4 (Ulk4) deletion as a rare copy number variation for a range of mental illnesses including schizophrenia (Lang et al., 2014). Deletion of Ulk4 in human neuroblastoma cells disrupted the composition of microtubules, which led to compromised neuritogenesis and cell motility. Using a gene knockdown approach mediated by *in utero* electroporation, we further demonstrated that silencing of Ulk4 in embryonic brain perturbed the proliferation and radial migration of NSCs during corticogenesis (Lang et al., 2016). Recently, Ulk4 hypomorph mice were created by deletion of Ulk4 exon 7 and DNA sequence afterward. Consistently, these mice exhibited perturbed neurogenesis at embryonic day 15.5 (E15.5) and a significantly thinner cerebral cortex at 12 days postnatal (Liu et al., 2016a). Our results and those of others strongly indicate that Ulk4 closely orchestrates brain development, which potentially connects Ulk4 deficiency with neurodevelopmental disorders.

However, it should be noted that the described RNA interference (RNAi) results can, at best, be used for the purpose of prediction, which may not recapitulate the scenario of physical gene downregulation. Although Liu et al. (2016a) have observed disorganized cortical lamination in Ulk4 hypomorph mice at 12 days postnatal, these phenotypes are very likely the direct outcome of hydrocephalus rather than the cause of aberrant brain formation. Therefore, the aforementioned results, especially those of *in vivo* studies, should be interpreted with great caution in the prediction of the roles of Ulk4 in corticogenesis.

In the present study, we have targeted Ulk4 exon 8 and created a hypomorphic mouse model and analyzed the process of corticogenesis at the early stage after birth. Strikingly, Ulk4 deletion did not affect the radial migration of pyramidal neurons, which was the main phenotype discovered in our previous RNAi study (Lang et al., 2016). In contrast to previous reports, our Ulk4 mice presented significantly thinner layers of II–III, and V, the most vulnerable regions for a range of neurodevelopmental disorders including SCZ. This layer-specific reduction was also confirmed in conditional Ulk4 deletion mice achieved by the Cre-loxp strategy. This abnormality may have been caused by decreased intermediate neural progenitors and increased apoptosis. Our data indicate that Ulk4 manipulates the behaviors of neural progenitors during brain development and, when functionally defective, leads to the reduction of specific cortical layers. This anomaly may increase predisposition to a range of neurodevelopmental disorders including SCZ.

## Materials and Methods

### Experimental Animals

**FIGURE 1:**
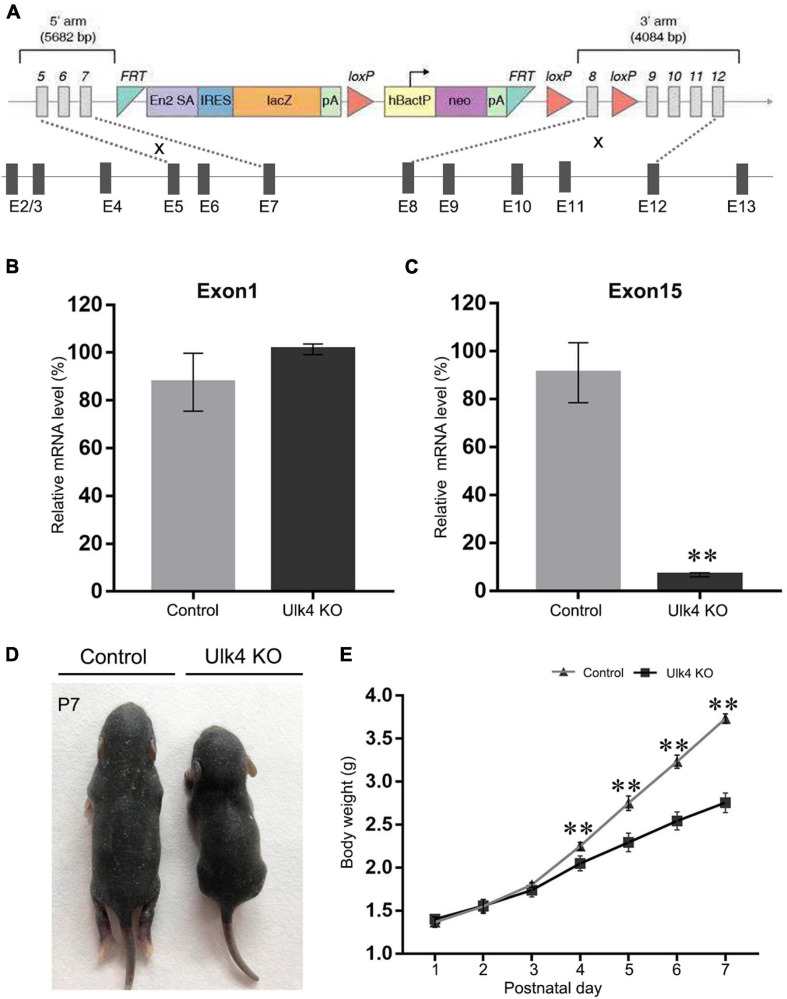
Creation and validation of Ulk4 knockout (KO) mice. **(A)** Diagram of the construct used to generate Ulk4 hypomorph mice. Note that the first seven exons were supposed to remain after successful homologous recombination. **(B,C)** Ulk4 mRNA expression was determined by RT-qPCR using two pairs of primers that recognize exons 1 and 15 of Ulk4. Compared to wild-type (WT) littermates, Ulk4 hypomorph mice exhibited comparable amounts of exon 1 **(B)** (control, 87 ± 13%; hypomorph, 101 ± 2%, *p* > 0.05, *n* = 4 each), but significantly low levels of exon 15 **(C)** (control, 91 ± 12%; KO, 6.6 ± 0.8%, **p < 0.01, *n* = 4 each, Student’ *t*-test), indicating a successful homologous recombination. **(D)** Ulk4 knockout mice presented a significantly reduced body size at postnatal day 7 (P7). **(E)** Ulk4 hypomorph mice displayed significantly lighter body weight than their littermates after P4, suggesting a marked growth retardation (*N* = 6 in each genotype). ^∗∗^*p* < 0.01, two-way repeated ANOVA.

The Ulk4^*tm*1a(KOMP)Wtsi^ sperm harboring a knockout-first construct was purchased from the Knockout Mouse Project (KOMP) Repository^1^ at the University of California, Davis. The knockout-first construct contained the sequence of FRT–En2SA–IRES–LacZ–PolyA–loxP–hbactP–neo–PolyA–FRT–loxP and was used to replace intron 7. This enables the production of a fused messenger RNA (mRNA) of Ulk4 exons 1–7 and FRT–En2SA–IRES–LacZ as the transcription terminates before the PolyA sequence. A further loxP was introduced to replace intron 8, which enables the generation of conditional Ulk4 knockout mice, if required. The detailed vector diagram is shown in Figure 1A. After *in vitro* fertilization, Ulk4 heterozygotes were generated on C57BL/6N strain background. Ulk4 depletion mice and control littermates were obtained from Ulk4^+/tm1a^ × Ulk4^+/tm1a^ mating. Each mouse was genotyped using genomic DNA with two pairs of primers. The wild-type (WT) allele was detected by a 304-bp DNA product using Ulk4-F (5′-TGTTGTCCAGGCCACTTCTTCC-3′) located in the 5′ arm and Ulk4-ttR (5′-TTCCCTGAAATAACCACACGGAGGG-3′) in exon 8. The primers for identifying the mutant allele were LoxF (5′-GAGATGGCGCAACGCAATTAATG-3′), which targeted the loxP site, and Ulk4-R (5′-ATTACTGTTGAGGTGCACACACTGC-3′) in the 3′ arm. A 234-bp DNA fragment was amplified from the mice with successful integration of the knockout-first construct.

The Ulk4 flox mice were obtained by Ulk4 heterozygote mating with Flp transgenic mice. Thus, the sequence between the two FRT sites was removed. Consequently, the eighth exon of Ulk4 is flanked by two loxP sites. To delete Ulk4 in neural stem cells, Ulk4^*fl/fl*^ mice were crossed with Nestin-Cre mice. Then, Nestin-Cre:Ulk4^*fl/fl*^ (referred to as Ulk4 conditional knockout, Ulk4^*Nestin*^ CKO) mice were generated. The date when the plug was found is considered to be E0.5. All experiments were performed in accordance with the Guidelines and Regulation of Laboratory Animals Used for Biomedical Studies of Shanghai, China. Animal care practices and all experiments were reviewed and approved by the Animal Committee of Department of Laboratory Science, Fudan University, Shanghai, China.

### Immunohistochemistry

Immunohistochemical staining was carried out as described previously (Song et al., 2016). Briefly, brains at various stages were harvested after fixation with 4% paraformaldehyde (PFA) overnight and sectioned in a cryostat at 20 μm. The following primary antibodies were used: TLE4 (1:300, mouse; SC365406, Santa Cruz), BrdU (1:1,000, rat; OBT0030G, Accurate), and PH3 (rabbit; 06-570, Upstate). The sections were incubated with primary antibodies overnight at 4°C, followed either by biotin-conjugated secondary antibodies (Vector Laboratories) for 3 h and then Cy3-conjugated streptavidin or with secondary antibodies conjugated to Alexa fluorochromes (Molecular Probes, Invitrogen). All slides were mounted with 75% glycerol containing nuclei counterstaining, Hoechst 33258 (Sigma, St. Louis, MO, United States). Images were captured with an epifluorescence microscope (Eclipse 80i, Nikon) equipped with software NIS-Elements F400.

### Bromodeoxyuridine Labeling Analysis

For bromodeoxyuridine (BrdU) pulse labeling, timed pregnant female mice at E13.5 or E15.5 received an intraperitoneal injection of BrdU (100 mg/kg body weight) 1 h prior to cervical dislocation. For the analysis of neuronal migration in the neocortex, pregnant mice were injected with BrdU at E13.5 or E15.5 and their offspring were killed at postnatal day 7 (P7). Brain sections were subject to citrate buffer-mediated antigen retrieval followed by antibody incubation, as described above.

### *In situ* Hybridization

*In situ* hybridization was performed as described in our previous studies (Lang et al., 2016). For the preparation of probes for Cux2, PlxnD1, Rorβ, Tbr2, and Pax6, total RNA was extracted from the cerebral cortex, and 400–600 bp cDNA fragments for each gene were subcloned into the PGEM-T vector (A362A, Promega), linearized for *in vitro* transcription, and subjected to DIG RNA labeling (SP6/T7, Roche Diagnostics Ltd., United Kingdom). The signals were visualized using 5-bromo-4-chloro-3-indolyl-phosphate (Boehringer Mannheim) and 4-nitroblue tetrazolium salt (BioRad) as substrates for alkaline phosphatase. Images of *in situ* hybridization were taken under the bright field of microscope (Eclipse 80i, Nikon). Detailed information of the used probes is listed in Supplementary Table 1.

### Terminal Deoxynucleotidyl Transferase-Mediated Nick-End Labeling Staining

Terminal deoxynucleotidyl transferase-mediated nick-end labeling (TUNEL) staining was performed as per the manufacturer’s instructions (Roche). Briefly, sections were incubated with permeabilization solution (0.1% Triton X-100 in 0.1% sodium citrate) for 30–60 min on ice. The reaction mixture containing 1 mM biotin-dUTP (11093070910, Roche) and 45 U terminal deoxynucleotidyl transferase (M1875, Promega) was applied to the sections for 1 h at 37°C, followed by incubation with Cy3-conjugated streptavidin (1:1,000; Sigma) and Hoechst 33258. Images were captured with an epifluorescent microscope (Eclipse 80i, Nikon) equipped with software NIS-Elements F400.

### RT-qPCR and Reverse Transcription PCR

Total RNA was isolated from P0 neocortex using Trizol according to the manufacturer’s instructions (Qiagen). Each sample was reverse transcribed using PrimeScript RT reagent kit with gDNA Eraser (RR047A, Takara).

Quantitative PCR (qPCR) was performed with SYBR green fluorescent master mix (Qiagen) on 7500 real-time PCR amplifier (Applied Biosystems). The sequences of the primers are listed as follows:

Forward for Ulk4 exon 1: GTCTTGTATGAGGAGA TTGGCAG
Reverse for Ulk4 exon 1: TTTCAGGCCGTTTGCACTTCT
Forward for Ulk4 exon 15: TAACTGGGATATAC GGTCCAAGG
Reverse for Ulk4 exon 15: TGTGATCGCCTCGA TAACAGG

For reverse transcription PCR (RT-PCR), regular PCR was performed with cDNA from the cerebral cortex of control and mutant mice. The sequences of the primers were as follows:

Forward for Ulk4 exon 4: CCAGAAGATGTCGTGAGAG
Reverse for Ulk4 exon 7: TGGCAATGGGTCTTCATAC
Reverse for Ulk4 exon 8: CAATCCGTCCAGCAAGTT

### Statistical Analysis

At least three animals of each genotype were used for statistical analysis to determine significance in each experimental paradigm. Student’s *t*-test was performed to compare differences between the two genotypes. For neuronal migration, the cortex was divided into 10 equal bins, spanning from the base of layer VI to the cortical surface, and the number of BrdU^+^ cells were counted by two separate researchers blind to the experiments. Two-way ANOVA was used to reveal any interaction between the genotype and the percentage of BrdU^+^ neurons in each bin. Cell counting and thickness measurements were conducted using ImageJ software, and statistical tests were run with GraphPad 8.0. All data are presented as the mean ± SEM. Differences were considered significant when *p* was less than 0.05.

## Results

### Ulk4 Deficiency Led to Growth Retardation

In the present study, Ulk4^*tm*1a^ sperms were acquired and underwent *in vitro* fertilization. Then, the fertilized eggs were transplanted into the uterus of pseudopregnant female mice. The offspring were genotyped (Supplementary Figures 1A,B) and Ulk4^*tm*1a^ heterozygotes were used to set up breeding pairs in order to produce Ulk4^*tm*1a/tm1a^ mice (with DNA sequence deletion after exon 7 of Ulk4, termed as Ulk4 hypomorph mice) and control littermates (Figure 1A). To further determine Ulk4 depletion efficiency, we compared the expression abundance of exons 1 and 15 by RT-qPCR. Our results showed that the mRNA abundance of exon 1 was comparable between the control and hypomorph mice (Figure 1B), whereas exon 15 mRNA abundance was sharply reduced by 90% in homozygotes (*p* < 0.001; Figure 1C) compared with that in control mice. We also performed RT-PCR to confirm the successful deletion of exon 8. A 439-bp band was amplified in both hypomorph and control mice when the primers targeting exon 4 (forward) and exon 7 (reverse) were used. On the contrary, no band was produced from hypomorph mice when the reverse primer targeting exon 8 was used, but a 500-bp band was amplified from control mice (Supplementary Figure 1C). We also performed immunostaining in P0 mouse brains and found that Ulk4 was strongly expressed in the cortex with relatively high intensity in layers II–V and the hippocampus (Supplementary Figures 2A,C), including CA1, CA3, and DG at P7 in control mice, whereas the expression was sharply decreased in Ulk4 hypomorph mice (Supplementary Figures 2B,D). Meanwhile, neonates of different genotypes were weighed on a daily basis. From P1 to P4, no body weight difference was found between the two genotypes. However, hypomorph mice became significantly lighter (Figure 1D) from P5, and their body sizes were much smaller than those of the littermates (Figure 1E). Over 90% mice could not survive to weaning. In contrast, Ulk4 heterozygotes did not display any obvious difference in body weight and size compared with WT mice (data not shown). This observation suggests that Ulk4 deficiency is detrimental for postnatal development and functional homeostasis of the brain.

### Ulk4 Deficiency Impaired Cerebral Cortex Development

**FIGURE 2:**
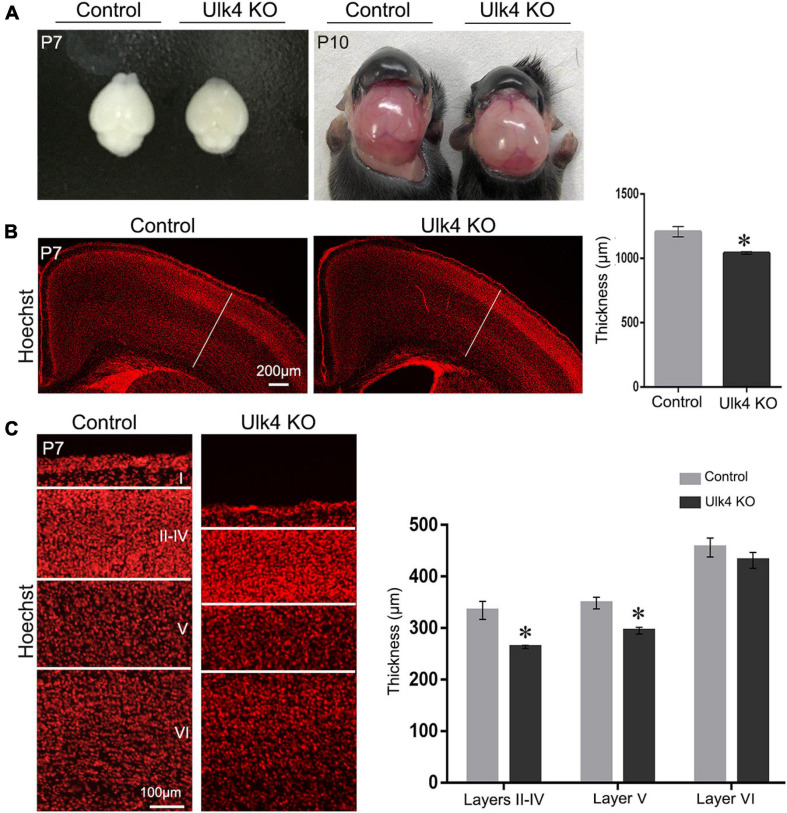
Ulk4 deficiency led to reduced thickness of the cerebral cortex. **(A)** Brain size of the control and hypomorph mice at postnatal day 7 (P7) and P10. Ulk4 hypomorph mice presented mildly smaller brains compared with their littermates at P7, but developed overtly dome-shaped heads at P10. **(B)** Hoechst staining revealed a significantly reduced thickness of the cerebral cortex in hypomorph mice in comparison to control littermates (*N* = 4 each). *p < 0.05, Student’s *t*-test. **(C)** Quantification of the thickness of the individual layers shown by Hoechst staining, revealing that layers II–V in hypomorph mice were significantly thinner than those in control mice (*N* = 4 each). *p < 0.05, two-way ANOVA. *Scale bars*, 200 μm in **(B)** and 100 μm in **(C)**.

Our previous studies have revealed that Ulk4 transcripts displayed a dynamic but extensive expression in neurogenic regions, ventricular (VZ) and subventricular (SVZ) zones, throughout the whole corticogenesis progress in mice. This expression could occur as early as E12.5 (Lang et al., 2016), coincident with the starting point of predominant cortical neurogenesis. Although most of the hypomorph mice developed severe hydrocephalus and present general reduction of the cerebral cortex at P10 (Figure 2A and Supplementary Figure 3), our longitudinal observation indicated that these mice still presented a gross normal brain size at P7 (Figure 2A) despite overt growth retardation (Figure 1E). We also evaluated gross cortical lamination of our mice at P7. Compared to control mice, the cortical thickness of mice with Ulk4 deletion was significantly reduced (Figure 2B). After a careful quantification of the individual layers shown by Hoechst staining, we found that this reduction occurred in layers II–V of the cortex as the thickness of layer VI was comparable (Figure 2C). Layer I was not measured and compared as the neurons originate from areas outside the VZ/SVZ of the dorsal cerebrum. We then surveyed the cortical thickness along the rostral–caudal axis and found widespread reduction in cortical thickness with no region-specific pattern (Supplementary Figure 4). Taken together, these results showed that Ulk4 deficiency affects the thickness of the cerebral cortex.

### Selective Reduction of Thickness of Layers II–III, and V in Hypomorph Mice

**FIGURE 3:**
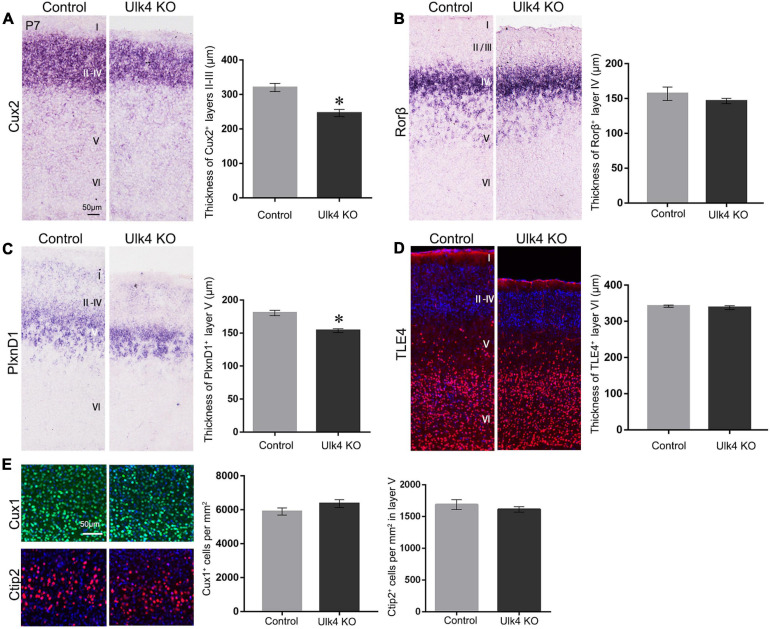
Selective reduction of the thickness of layers II–III, and V in hypomorph mice. **(A)** Coronal sections from postnatal day 7 (P7) brains stained with various layer-specific markers. Cux2 *in situ* hybridization showed the decreased thickness of layers II–IV in hypomorph mice **(A)** (*p < 0.05) compared with control mice. **(B)** *In situ* hybridization of Rorβ, densely expressed in layer IV, revealed no significant difference in the thickness between hypomorph and control mice. **(C)** The thickness of layer V was significantly reduced in hypomorph mice, as shown by *in situ* hybridization of Plxnd1, a marker of layer V (*p < 0.05). **(D)** Anti-TLE4 staining did not show any changes in the thickness of layer VI in hypomorph mice, consistent with the quantification in Hoechst staining. **(E)** The cell density in layers II–V was unaltered in our mice, as shown by the immunostaining with Cux1 (layers II–IV, *green*) and Ctip2 (layer V, *red*). *N* = 4 each, Student’s *t*-test. *Scale bar*, 50 μm.

To precisely pinpoint the affected cortical layers of our mice, we used layer-specific markers to decipher the cortical laminar organization in both Ulk4 hypomorph mice and control littermates. The transcription factor Cux2 was commonly used as a marker for layers II–IV. The results showed a dramatic decrease in the thickness of the Cux2^+^ layers in hypomorph mice (Figure 3A). We also carried out *in situ* hybridization of Rorβ, which is densely expressed in layer IV, but did not find any obvious difference there (Figure 3B). Thus, among the upper layers, the thickness of layers II–III, but not layer IV, is reduced in hypomorph mice.

To determine whether Ulk4 deficiency affected the formation of layer V, we conducted *in situ* hybridization for PlxnD1, expressed by callosal projection neurons in layer V. The results revealed that the thickness of the PlxnD1^+^ layer was significantly reduced in hypomorph mice (Figure 3C). Finally, we performed anti-TLE4 staining and confirmed that there was no significant reduction in the thickness of layer VI in hypomorph mice (Figure 3D). However, the density of cells in layers II–V was unaltered in our mice, as shown by immunostaining with Cux1 (layers II–IV) and Ctip2 (layer V) (Figure 3E). These results indicate that Ulk4 deletion specifically targets the development of layers II–III, and V.

### Cortical Neuron Migration Is Not Affected in Ulk4 Hypomorph Mice

Reduced cortical sublayers may be the result of dysfunctional radial migration, a fundamental process essential for embryonic brain lamination. A “delayed” radial migration was observed in Ulk4-knockdown cortex (Lang et al., 2016). To address this, we performed “birth-dating” experiments and analyzed radial migration as previously reported (Keays et al., 2007) with our mice.

**FIGURE 4:**
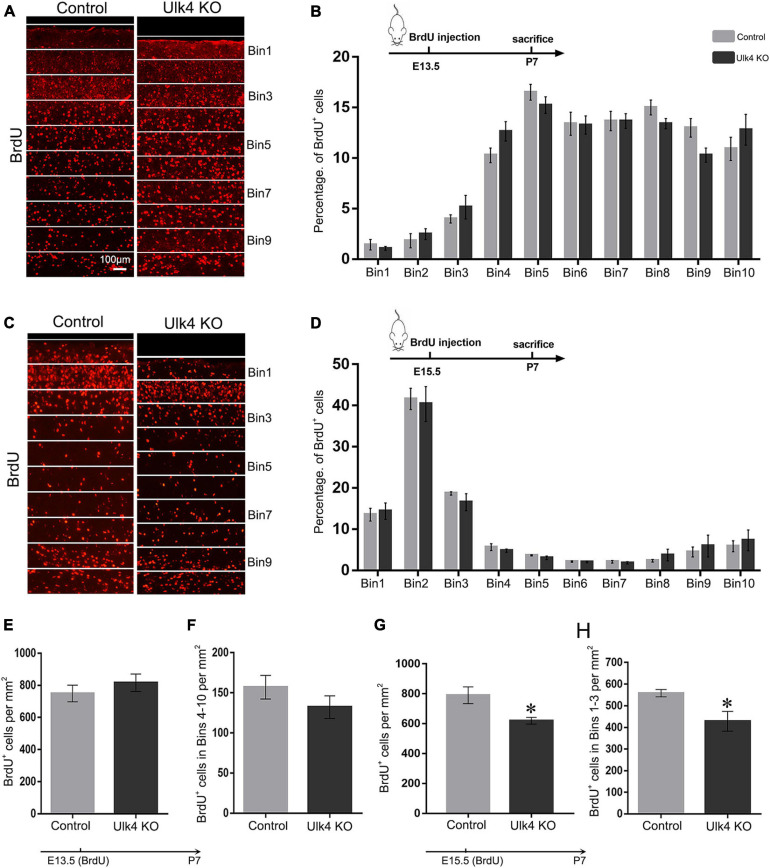
Cortical neuron migration is not affected in Ulk4 hypomorph mice. **(A–D)** Pregnant mice received an intraperitoneal injection of BrdU at embryonic day 13.5 (E13.5) **(A,B)** or E15.5 **(C,D)** and the brains were harvested at postnatal day 7 (P7). The cortex was divided into 10 equal bins **(A,C)**, spanning from the intermediate zone to the molecular layer. BrdU^+^ cells were counted blind to the genotypes. The mean percentage of BrdU^+^ cells in bins 1–10 was quantified for the control and hypomorph mice **(B,D)**. No association between the genotype and the distribution of cells across the bins was revealed [two-way ANOVA, E13.5: *F*_(9, 54)_ = 1.5, *p* > 0.05; E15.5: *F*_(9, 198)_ = 0.25, *p* > 0.05, *N* = 5 each]. *Scale bar*, 100 μm. **(E,F)** No significant difference was detected in the number of BrdU^+^ cells in the whole cerebral cortex **(E)** or bins 4–10 **(F)** between hypomorph and control mice when BrdU was given at E13.5. *N* = 4 each, *p* > 0.05, Student’s *t*-test. **(G,H).** However, the numbers of BrdU^+^ cells were significantly reduced in the whole cortex and bins 1–3 in hypomorph mice compared with the control mice (^∗^*p* < 0.05, Student’s *t*-test) when BrdU was given at E15.5.

As the migration of cortical neurons follows an inside-out fashion, in which early born and late-born neurons are located in deep and upper cortical layers, respectively, we gave a pulse BrdU injection to pregnant mice at E13.5 or E15.5, the two “time windows” when most of the neurons in the upper layers and deep layers were generated, respectively. The offspring brains were collected at P7 and their cortices were divided into 10 equal bins (Keays et al. 2007). The number of BrdU^+^ neurons in each bin was calculated and the percentage was compared. In the brains that received BrdU injection at E13.5, most of the BrdU^+^ neurons were located in bins 4–10, with very few in bins 1–3 (Figure 4A). Statistical analyses did not reveal any genotype-dependent difference in the cell percentage of each bin (Figure 4B) or any significant difference in the number of BrdU^+^ neurons in bins 4–10 and the whole cortex (Figures 4E,F). This finding indicates that the migration and survival of neurons located in the deep layer were not affected in our mice.

We performed similar studies with the brains that received a pulse BrdU injection at E15.5. Over 70% of the BrdU^+^ neurons were located in bins 1–3, presumably corresponding to layers II–IV, and the rest of the cells were sparsely located in the deeper layers of the cortex in both groups (Figure 4C). Although no significant difference in the percentage of BrdU^+^ neurons in each bin was detected (two-way ANOVA: *p* = 0.15; Figure 4D), hypomorph mice presented significantly decreased cell numbers of BrdU^+^ neurons in the whole cortex as well as in bins 1–3 (two-way ANOVA: *p* = 0.03; Figures 4G,H). This finding strongly suggests that, although the radial migration of neurons born at E15.5 of hypomorph mice is not overtly perturbed, they may have undergone either less active proliferation in the neurogenic regions or abnormal cell death once the migration was completed.

### Abnormal Cell Death in Cortical Superficial Layers of Hypomorph Mice

**FIGURE 5:**
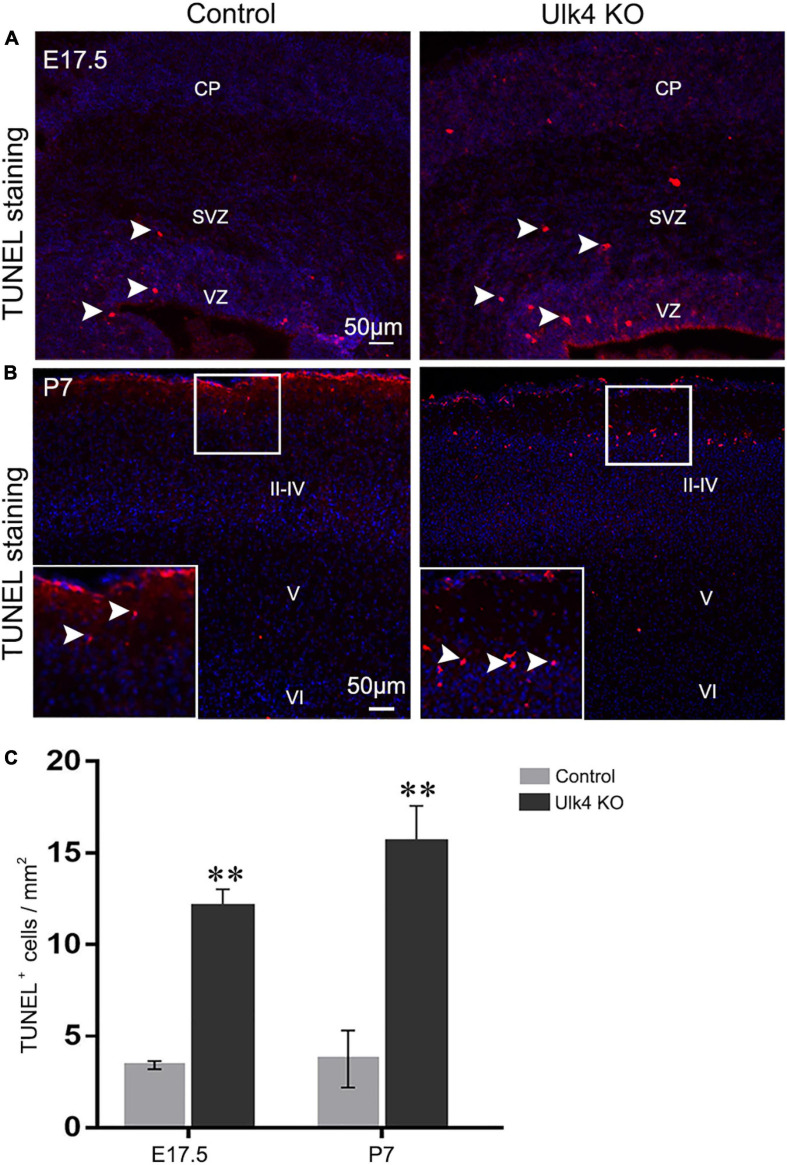
Abnormal cell death in the cortical layers of hypomorph mice. **(A–C)** Hypomorph mice presented more TUNEL^+^ cells (*red*, *white arrowed*) at E17.5 **(A)** and in layers II–III of P7 cortex **(B)**. *VZ*, ventricular zone; *SVZ*, subventricular zone; *CP*, cortical plate. *N* = 3 each. **p < 0.01, two-way ANOVA. *Scale bar*, 50 μm.

To explore the possibility that the reduction of cortical thickness is caused by abnormal cell death in hypomorph mice, TUNEL staining was performed at E15.5, E17.5, and P7. No obvious differences were found in the number of TUNEL^+^ cells in the cortex of the control and hypomorph mice at E15.5 (data not shown). Interestingly, a drastic increase of TUNEL^+^ cells was found in the VZ, SVZ, and the cortical plate of hypomorph mice at E17.5 (two-way ANOVA: *p* = 0.006; Figures 5A,C) and P7 (two-way ANOVA: *p* = 0.009; Figures 5B,C). Importantly, the TUNEL^+^ cells at P7 cortex were mainly observed in layers II–III, and there was no salient cell death in layers IV–VI (Figure 5B). Therefore, we conclude that increased cell death might account for the decreased neuron number and reduced thickness of the cortical layers, particularly for layers II–III.

### The Intermediate Progenitors Are Decreased at E15.5 in Hypomorph Mice

**FIGURE 6:**
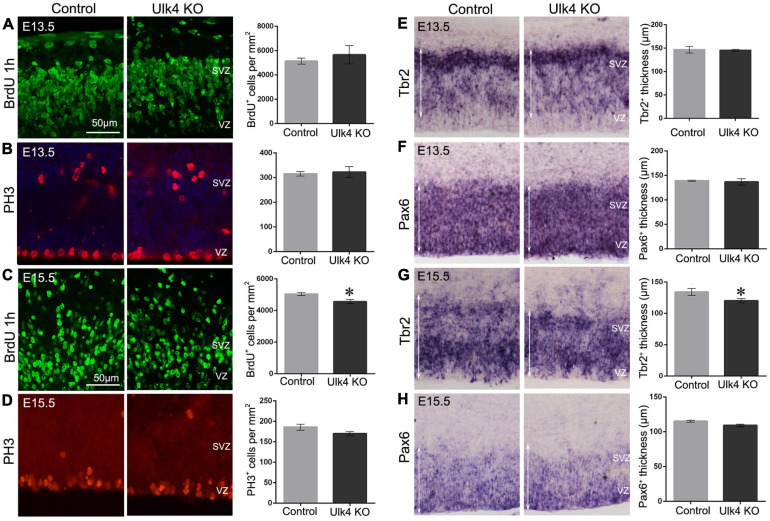
Hypomorph mice had a reduced intermediate progenitor pool at embryonic day 15.5 (E15.5). **(A–D)** Immunostaining of BrdU (*green*) and PH3 (*red*) at E13.5 **(A,B)** or E15.5 **(C,D)**. No difference was detected in the numbers of PH3^+^ cells at both E13.5 and E15.5 (*p* > 0.05, Student’s *t*-test). However, a decreased number of BrdU^+^ cells was found at E15.5 (**C**) (*N* = 5 each, ^∗^*p* < 0.05, Student’s *t-*test), but not at E13.5 **(A)** (*N* = 3 each, *p* > 0.05, Student’s *t*-test). **(E–H)** *In situ* hybridization of Pax6 **(F,H)** and Tbr2 **(E,G)** at E13.5 **(E,F)** and E15.5 **(G,H)**. There was about 10% decrease in the thickness of the Tbr2^+^ ventricular (VZ)/subventricular (SVZ) regions (indicated by *white arrows*) at E15.5 in hypomorph embryos **(G)** (*N* = 5 each, ^∗^*p* < 0.05, Student’s *t*-test), whereas no significant difference was detected at E13.5 **(E)** (*N* = 3 each, *p* > 0.05, Student’s *t*-test). Pax6 expression in VZ/SVZ did not differ significantly at E15.5 **(H)** (*N* = 3 each, *p* > 0.05, Student’s *t*-test) or E13.5 **(F)** (*N* = 3 each, *p* > 0.05, Student’s *t*-test) in hypomorph mice relative to the control mice. *Scale bar*, 50 μm.

Decreased proliferation of neural stem cells in the neurogenic regions might also contribute to the thinned cortical layers in our mice. To address this, the proliferative capacity of neural progenitors was examined 1 h after BrdU injection. At E13.5, the number of BrdU^+^ cells appeared to be similar between the two genotypes (Figure 6A), which was confirmed by immunostaining with anti-phosphorylated histone 3 (Figure 6B), a more specific marker for proliferating progenitors in late G2 and M phases. Pax6 and Tbr2 are two transcription factors that determine the commitment of apical/basal progenitors in the VZ and SVZ (Englund et al., 2005). We then performed hybridization for Pax6 (Figure 6F) and Tbr2 (Figure 6E), but did not find any difference between hypomorph and control brains. Thus, it is likely that the proliferation of neural stem cells is unaffected in our mice at E13.5.

However, at E15.5, the number of BrdU-labeled progenitors was significantly reduced in hypomorph mice (two-way ANOVA: *p* = 0.024; Figure 6C), indicating a decreased proliferation of neural projectors at this stage. The number of PH3^+^ cells also show a declining tread in hypomorph brains, although there was no statistical difference (Figure 6D). Furthermore, Tbr2^+^ intermediate neural progenitors were also reduced in number, as evidenced by about 10% decrease in the thickness of the Tbr2^+^ region (*p* = 0.02; Figure 6G). In contrast, Pax6 expression in the VZ did not differ significantly at E15.5 in hypomorph mice relative to the controls (Figure 6H). These results suggest that Ulk4 might play a role in the proliferation of neural progenitors in the neurogenic region at E15.5.

### Targeted Deletion of Ulk4 in Neural Stem Cells Recapitulates the Phenotype of Thinner Cortex in Conditional KO Mice

Our results strongly indicate that conventional depletion of Ulk4 alters the property of neural stem cells and results in abnormal cell death, which may underpin the observed reduction of the cortical layers. However, it is not clear whether this observed abnormality has any connection with congenital hydrocephalus, which robustly disrupts the cell cycle (Owen-Lynch et al., 2003) and inhibits the proliferation of neural stem cells. To address this question, we deleted Ulk4 specifically in neural stem cells and produced Ulk4 conditional knockout (Ulk4^*Nestin*^ CKO) mice.

**FIGURE 7:**
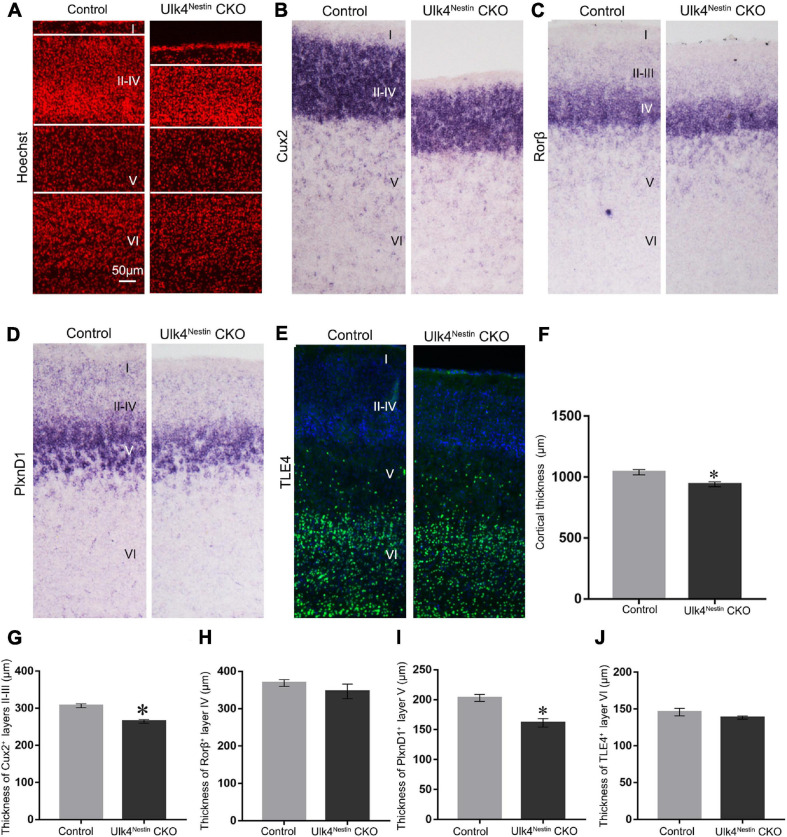
Targeted deletion of Ulk4 in neural stem cells recapitulates the phenotype of thinner cortex in conditional knockout (CKO) mice. **(A–J)** Similar to the phenotypes observed in hypomorph mice, staining using layer-specific markers showed that the thickness of layers II–III, and V was also reduced in the cortex of Ulk4^*nestin*^ CKO mice, as shown by Hoechst **(A,F)** and Cux2 **(B,G)** staining and plxnD1 expression **(D,I)**, whereas the thickness of Rorβ^+^ layer IV **(C,H)** and TLE4^+^ layer VI **(E,J)** was unaltered. *N* = 4 each, ^∗^*p* < 0.05, Student’s *t*-test. *Scale bar*, 50 μm.

As expected, Ulk4^*Nestin*^ CKO mice can survive into adulthood with no preweaning or postnatal loss (Supplementary Figures 5A,B and Supplementary Table 2). They did not have the typical dome-shaped head or weight difference compared to control littermates. Nissl staining also revealed a clear cortical lamination (Supplementary Figures 5C,D). We then performed layer-specific staining to compare the cortical lamination between CKO and control mice. Consistently, CKO mice displayed thinner cerebral cortex (Figures 7A,F), specifically layers II–III, and V, as revealed by staining with Cux2 and PlxnD1 (Figures 7B,D,G,I), whereas the thickness of layers IV and VI (Rorβ^+^ and TLE4^+^ staining) was unaltered at P7 (Figures 7C,E,H,J). These data strongly suggest that the described phenotypes in Ulk4 hypomorph mice are largely caused by perturbed brain development.

## Discussion

SCZ is a chronically disabling brain disorder characterized by psychotic symptoms, such as positive and negative symptoms, abnormal social behaviors, and cognitive impairment. Although a vast body of risk loci has been proposed by recent genetic research, only a handful of them are replicated by independent cohort validation and underwent stringent validation by functional and animal studies. After analyzing two big databases (International Schizophrenia Consortium and Decode), we have reported that recurrent deletion of Ulk4 co-segregates a spectrum of neurodevelopmental disorders including autism and SCZ. A recent study of the Brain and Body Genetic Resource Exchange (BBGRE) cohort also reported a morbidity of 1.2‰ population, displaying ULK4 copy number variations and exhibiting pleiotropic neurodevelopment problems including learning difficulty and language delay. Ou et al. (2019) revealed that Ulk4 polymorphism is significantly associated with autism in a Chinese population and the expression of Ulk4 transcripts in postmortem human prefrontal cortex. These genetic findings strongly highlight a fundamental role of Ulk4 during brain formation and maturation. In this study, using “conventional” and “conditional” gene loss-of-function approaches, we have demonstrated that Ulk4 regulates NSC proliferation and the survival of progeny cells in a layer-specific manner. Deletion of Ulk4 selectively disrupts the cellular architecture of layers II–III, and V in the cerebral cortex, which may predispose the occurrence of many neurodevelopmental disorders.

Ulk4 is a member of the Unc-51-like serine/threonine kinase family (Lang et al., 2014). Previously, we have shown that Ulk4 depletion inhibits cell cycle progression in cultured human neuroblastoma cell line (SH-5YSY) (Lang et al., 2014) and Ulk4-knockdown brains (Lang et al., 2016). In line with this observation, Liu et al. (2016a) found that Ulk4 was actively expressed in dividing SH-5YSY cells and peaked in the G2/M phases, but less in division-quiescent cells. Both the mice in Liu et al. (2016a) and our mice exhibited a significantly reduced BrdU^+^ incorporation at E15.5, and we further demonstrated that this was in coincidence with the reduced *in situ* signals of Tbr2^+^ cells, progenitors contributing to the generation of neurons in superficial layers. These data strongly indicate that Ulk4 may actively regulate the cell cycle progression of NSCs and precisely manipulate their number during corticogenesis.

It remains unclear how Ulk4 preferably regulates the proliferating capacity of NSCs in a time- or layer-specific manner, as no reduction of BrdU^+^ cells in our Ulk4 hypomorph mice was detected at E13.5. Results from whole-genome sequencing using Ulk4 hypomorph brains at P14 have demonstrated a complex transcriptome picture, among which the 618 most relevant molecules were enriched in “neural precursor cell proliferation” and “cell cycle” signaling pathways (Liu et al., 2016a). Thus, the behaviors of NSCs with Ulk4 deficiency were synergically under control of many genetically programmed pathways. Wnt signaling is well documented to orchestrate key events of NSCs, including “stemness” maintenance (Chenn, 2008), activity switch from proliferation to migration (Ishizuka et al., 2011), neuronal commitment (Freese et al., 2010; Munji et al., 2011), and neural circuit assembly (Salinas and Zou, 2008; Bocchi et al., 2017). Notably, many Wnt signaling elements including Apc, Fzd10, Fzd2, Fzd6, Lef1, Porcn, Sox17, etc., and pathways that crosstalk with canonical/non-canonical Wnt/β-catenin signaling (such as Notch, FGF, BMP, HH, etc.) displayed altered expression profiles in P14 Ulk4 hypomorph mouse brains (Liu et al., 2016a). For example, adenomatous polyposis coli (Apc) is a tumor suppressor gene that regulates nuclei β-catenin and TCF/LEF signaling. Conditional depletion of Apc led to the defective generation and migration of neurons and severely disrupted cortical lamination (Yokota et al., 2009). Similarly, overexpression of Fzd6, one of the Wnt receptors, was strongly associated with aggressive growth of neuroblastoma and poor patient survival (Cantilena et al., 2011).

In addition, Fgfr2 and EphB1 are two important proteins that interconnect with Wnt signaling and, when downregulated, caused a reduction of the NSC pool (Zheng et al., 2004; Chumley et al., 2007). Interestingly, they also showed downregulated expressions in hypomorph brains. On the other hand, unbalanced Wnt signaling promotes premature cell cycle exit (Chenn and Walsh, 2002) and cell death (Lo Sardo et al., 2012) and determines the thickness and surface area of the cortex. In our mice, increased apoptosis could be detected as early as E17.5 (Figure 5A), a key developmental time window when the ventricles were not ciliated, and culminated at P7 (Figure 5A), when the multi-ciliated ependymal cells are supposed to appear (Banizs et al., 2005). Recently, the structure of Ulk4 was resolved and interacting partners were reported, including tubulin binding and centrosomal proteins (Khamrui et al., 2020; Preuss et al., 2020), such as HAUS2 and HAUS8 (Uehara et al., 2009), CCP110 (centriolar coiled-coil protein 110), CEP97 (centrosomal protein 97), CSPP1 (centrosome and spindle pole-associated protein 1), and OFD1 (OFD1 centriole and centriolar satellite protein), and motor proteins of the kinesin family (KIF1B, KIF3B, and KIFAP3). These proteins were widely involved in the cell cycle, cell polarity, and cytoskeleton arrangement, highlighting the role of Ulk4 in cell division and migration of neuronal progenitors.

Radial migration is probably the most complex process to ensure correct “nesting” of post-mitotic cells and to support the formation of a multilayered cerebral cortex. The precise regulatory elements are yet to be fully clear, but functional integrity of radial glia is essential. How Ulk4 is involved in this process remains considerably arguable, and current evidence is mixed. We have previously discovered intense *in situ* signals of Ulk4 mRNA in the VZ/SVZ regions of mouse brains (Lang et al., 2016). This finding is further confirmed by the co-expression of Ulk4 with SOX3 and BLBP, two markers of radial glia, throughout the developing *Xenopus* brain (Dominguez et al., 2015). In Ulk4-knockdown brains, a massively delayed radial migration was observed when Ulk4 shRNA268 was introduced, and this could be successfully reversed after a forced Ulk4 overexpression. However, a second shRNA269 only caused a sparsely packed cell population in the individual sublayers with no obvious migration delay (Lang et al., 2016). Liu et al. (2016a) reported thinner layers II and III and cortices as a whole in P14 Ulk4 hypomorph mice. However, it is plausible to connect this reduction with defective migration if considering the influence of congenital hydrocephalus (Liu et al., 2016b). Indeed, hydrocephalus may either have “add-on” effects or “mask” the genuine migration delay through resultant thinning of the individual sublayers. In the present study, the cortices were divided into 10 bins with equal width, and the migration trajectory of post-mitotic neurons born at E13.5 and E15.5 were tracked at P7. Interestingly, no genotype-relevant migration abnormality was detected in each bin, which hints that the reduced cortical layers are more likely the results of defective cell proliferation and abundant cell death. Nevertheless, this still remains an interesting debate, and a properly designed exploration in Ulk4 conditional knockout mice may provide a clearer explanation.

It should be noted that Ulk4 was recently proposed as a ciliopathy-relevant gene, as Ulk4 deletion unanimously causes severe congenital hydrocephalus in mice, which is often associated with a disarranged axoneme assembly and disrupted function of motile cilia (Vogel et al., 2012; Liu et al., 2016b). In clinics, patients with Ulk4 variants mostly manifest symptoms featuring abnormal neurodevelopment and cognition (Lang et al., 2014; Liu et al., 2016a). However, the relationship between Ulk4 and ciliopathy is an attractive research field, and further follow-up studies need to be warranted.

Detailed molecular pathways are yet to be established, but Ulk4 hypomorph mice displayed dysregulated inhibitory circuits (Liu et al., 2018a) and reduced white matter integrity (Liu et al., 2018b), the two most frequent phenotypes that predispose mental disorders and neurodevelopmental diseases (Lewis et al., 2005; Peters and Karlsgodt, 2015). Unbalanced Wnt signaling is another promising candidate that links the two categories of diseases. Patients with schizophrenia and bipolar disorder often display attenuated canonical Wnt signaling and enhanced non-canonical signaling, particularly the Wnt/Ca2^+^ pathway (Hoseth et al., 2018). This functional switch can be facilitated by primary cilia (Basten and Giles, 2013). How Ulk4 is involved in this process remains unknown, but, as aforementioned, Ulk4 modulates the transcription of many key elements of the Wnt signaling pathway and the expression of at least 66 ciliogenesis-relevant genes (Liu et al., 2016a). Therefore, more direct evidence shall be pursued in future follow-up studies.

## Data Availability Statement

The original contributions presented in the study are included in the article/Supplementary Material, further inquiries can be directed to the corresponding author/s.

## Ethics Statement

The animal study was reviewed and approved by the Animal Committee of Department of Laboratory Science, Fudan University, Shanghai, China.

## Author Contributions

BL and Y-QD designed the research. N-NS, J-YC, YH, and Y-LS generated the mice. LH, YC, and C-PY carried out the experiment and analyzed the data. Y-QD, BL, and LH wrote the manuscript. All authors read and approved the final manuscript.

## Conflict of Interest

The authors declare that the research was conducted in the absence of any commercial or financial relationships that could be construed as a potential conflict of interest.

## Footnotes

1 www.KOMP.org
