# Supplementary material for: Ulk4, a Newly Discovered Susceptibility Gene for Schizophrenia, Regulates Corticogenesis in Mice
Source: Front Cell Dev Biol. 2021 Jun 21;9:645368. doi: 10.3389/fcell.2021.645368 (PMC8255617; doi:10.3389/fcell.2021.645368)
Supplement: Supplementary file 1 [file Data_Sheet_1.docx]

# Supplementary Fig1. Genotyping of neonatal offspring and confirmation of targeting sequence in hypomorph mice. (A) Showing the diagram of the targeting vector and the location of used primers. (B) LoxF/ULK4-R primers were used to detect the targeting band while ULK4-F/ ULK4-ttR was used to detect endogenous band. The product size of LoxF/ULK4-R was 234bp and of ULK4-F/ULK4-ttR was 304bp. From the left to right, the genotypes of the offspring were: 1, wild type; 2, heterozygote; 3, homozygote; 4, heterozygote. (C) PCR were performed with the cDNA extracted from control and hypomorph mice to confirm that the deleted DNA sequence. A 439bp band was detected in both Ulk4 hypomorph and control mice when the forward primer targeted exon4 and reverse primer recognized exon7 whereas no band was found in mutant mice with the reverse primer targeted exon8 in contrast to a 500bp band in control mice.

# Supplementary Fig2. Validation of Ulk4 mutant mice. (A-B) The immunostaining results showed that Ulk4 was widely expressed in the cortex with relatively high intensity in the layer II-V and hippocampus including CA1, CA3 and DG at P7 whereas the expression was sharply decreased in Ulk4 mutant mice. Scale bars=100μm.

**Supplementary Fig3. The structure of cerebral cortex in the mutant mice at P0 and P10.** (A-C) Nissl staining showed that the KO mice retained six-layer structure and the lateral ventricles was wildly enlarged at P0. (A). The mutant mice presented enlarged lateral ventricles and reduced cortex compared with control littermates at P10 (B). Scale bars=400μm. Wm, white matter; Ctx, cerebral cortex; LV, lateral ventricles; Se, septum; St, striatum;

#

# Supplementary Fig4. Ulk4 mutant mice present unanimous reduction in the cortical thickness spanning from the rostral to caudal at P7. (A-C) The thickness of cerebral cortex was decreased by 7%, 13% and 15% in the motor cortex (A), somatosensory cortex (B) and visual cortex (C) of KO mice, respectively, compared with control. The white line indicated the location for measurement. The left panel was 4x image with a scale bar of 200μm while the middle panel was 10×images with a scale bar of 100μm as shown in images. The right panel shows the statistical results. N=3 each. Ctx, cortex; Hi, hippocampus. I-VI, different sublayers of cortex. *, *p*<0.05. Student’s *t*-test.

**Supplementary Fig5. Ulk4^Nestin^ CKO mice present relatively intact structure of cerebral cortex.** (A) Five litters of pups (40 in total) were analyzed and 7 Ulk4 conditional knockout mice were identified. The ratio fits 1/8 progeny prediction based on Mendel's law of inheritance. (B) The mice were weighed at P0, P6and 2 month and no significant difference in weight was found at these stages between control and CKO mice. (C, D) Compared with wildtype littermates, Ulk4^Nestin^ CKO mice retained six-layer structure in the cortex albeit enlarged lateral ventricles at both P7 (C) and 3 months (D). Scale bars=400μm. Cc, corpus callosum; Ctx, cerebral cortex; LV, lateral ventricles; Se, septum; St, striatum.

**Supplementary Fig6**. The detailed co-ordinates of the exon/intron 1-9 of mouse Ulk4 in chromosome 9 (Build GRCm38).
