# Supplementary material for: Ulk4, a Newly Discovered Susceptibility Gene for Schizophrenia, Regulates Corticogenesis in Mice
Source: Front Cell Dev Biol. 2021 Jun 21;9:645368. doi: 10.3389/fcell.2021.645368 (PMC8255617; doi:10.3389/fcell.2021.645368)
Supplement: Supplementary file 2 [file Table_1.DOCX]

**Table S1.** Detailed Information About the Probes Used for In Situ Hybridization

| **Gene name** | **GenBank ID** | **Forward Primer** | **Reverse primer** | **Amplicon size** |
| --- | --- | --- | --- | --- |
| Cux2 | NM_001312908.1 | TCCTCCAGCTACTCCGGAC | TGGAGTATGTGTCCAGCTC | 733 |
| Rorβ | NM_001289921.1 | AGCCTGTATGCTGAGGTGCA | AAGATCTGATCGTTCTGACACA | 615 |
| Pax6 | NM_001244198.2 | GGGAGTGCCCTTCCATCT | CCCATGGGCTGACTGTTC | 866 |
| Tbr2 | NM_010136.3 | GACATCGGTTCTTATGAATCTG | ATGAATCAATCCAGCACCTT | 728 |
| PlxnD1 | NM_026376 | CAGGAAATGAACGCACACC | TGAGGGACACAGACAACT | 651 |
