# Supplementary material for: Ulk4, a Newly Discovered Susceptibility Gene for Schizophrenia, Regulates Corticogenesis in Mice
Source: Front Cell Dev Biol. 2021 Jun 21;9:645368. doi: 10.3389/fcell.2021.645368 (PMC8255617; doi:10.3389/fcell.2021.645368)
Supplement: Supplementary file 3 [file Table_2.DOCX]

**Table S2. Detailed information of genotyping pattern for the breeding pairs of Nestin-cre: Ulk4^+/-^ X Ulk4^+/-^**

| Date of Birth | Total number | Heterozygote | CKO |
| --- | --- | --- | --- |
| 2017-6-12 | 12 | 1 | 2 |
| 2017-6-13 | 6 | 4 | 1 |
| 2017-8-12 | 8 | 0 | 1 |
| 2019-1-10 | 7 | 0 | 1 |
| 2019-1-12 | 7 | 2 | 2 |
