# Supplementary figures and images for: Ulk4, a Newly Discovered Susceptibility Gene for Schizophrenia, Regulates Corticogenesis in Mice
Source: Front Cell Dev Biol. 2021 Jun 21;9:645368. doi: 10.3389/fcell.2021.645368 (PMC8255617; doi:10.3389/fcell.2021.645368)

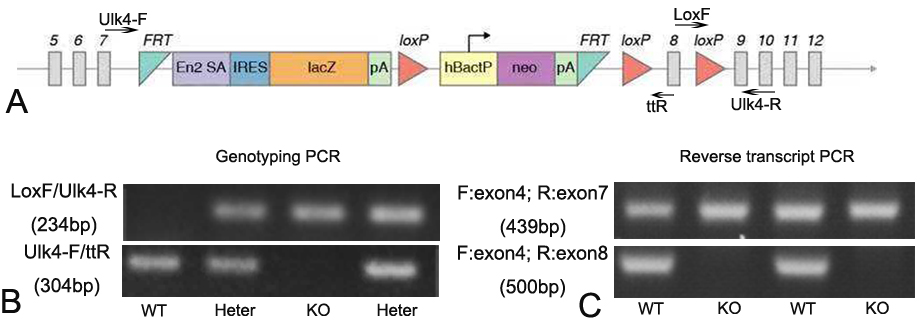

Supplement: Supplementary file 4 [file Image_1.JPEG]

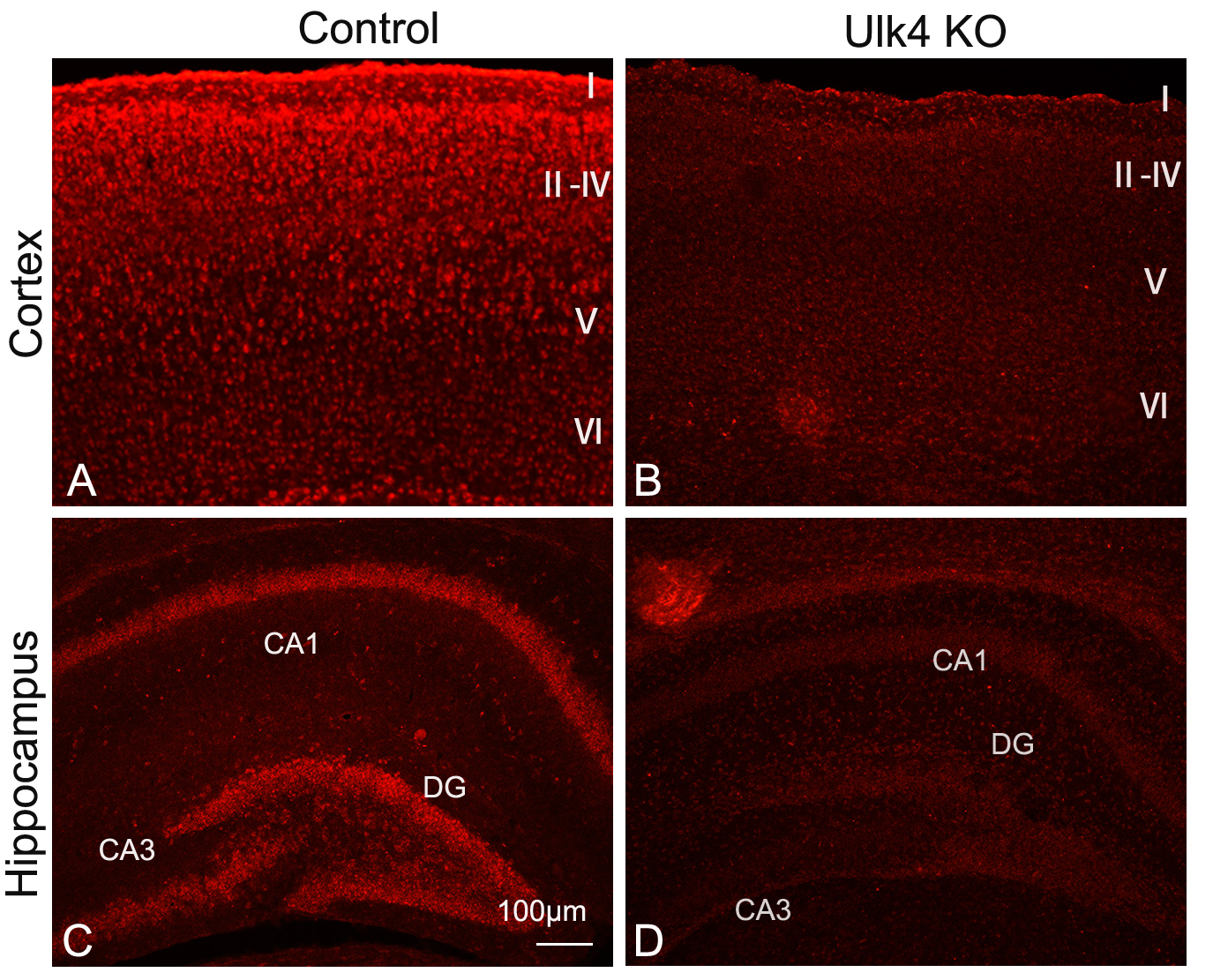

Supplement: Supplementary file 5 [file Image_2.JPEG]

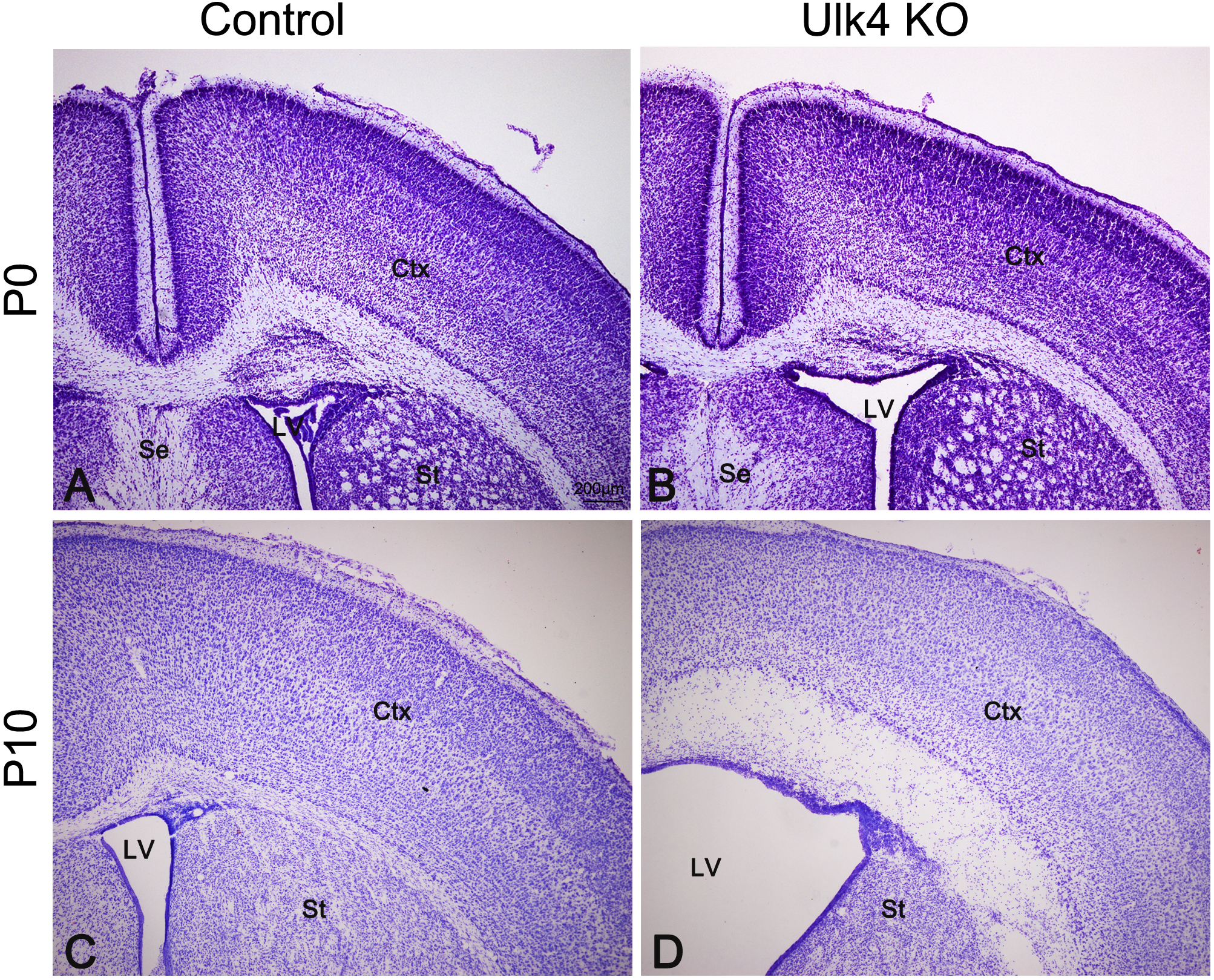

Supplement: Supplementary file 6 [file Image_3.JPEG]

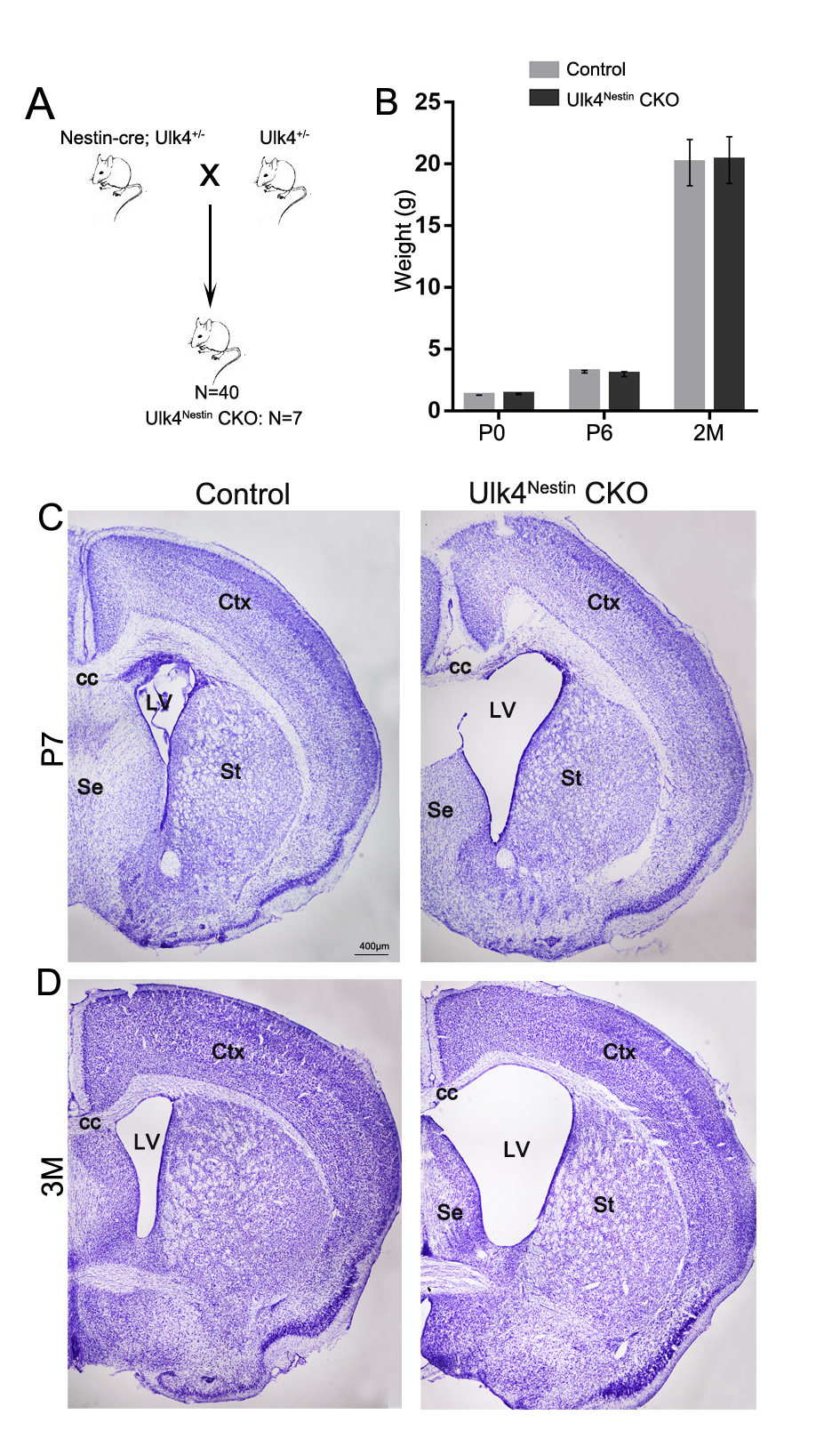

Supplement: Supplementary file 7 [file Image_4.JPEG]

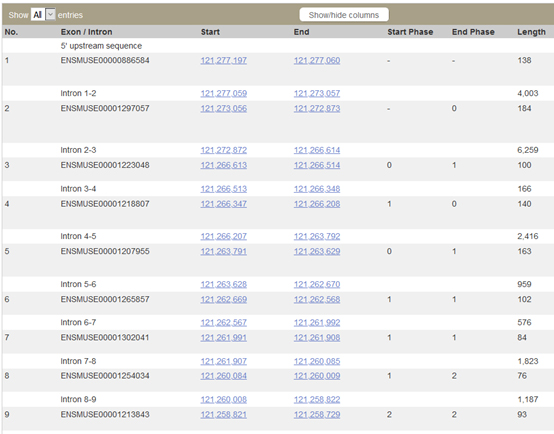

Supplement: Supplementary file 8 [file Image_5.JPEG]

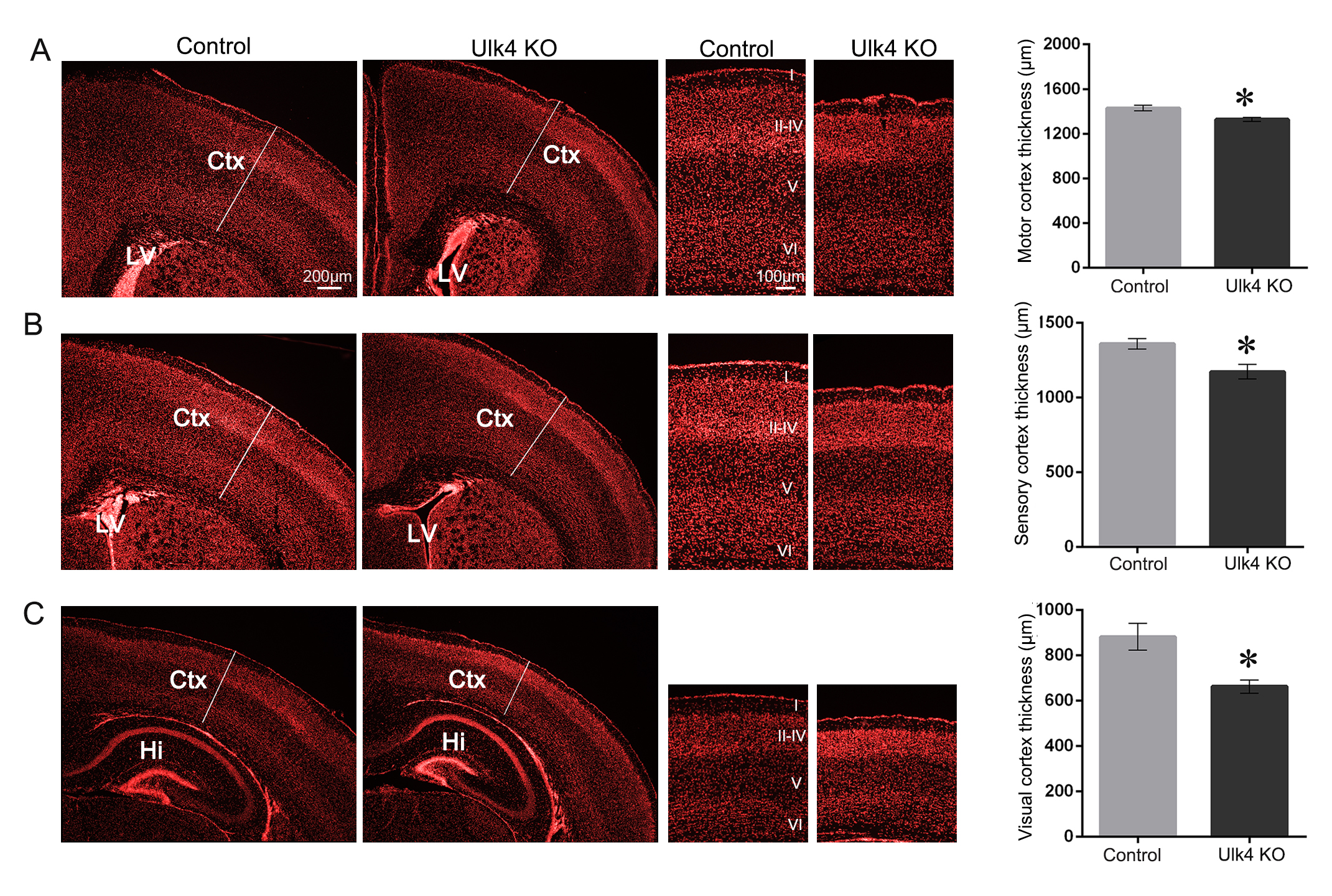

Supplement: Supplementary file 9 [file Image_6.JPEG]
